# Draft genome sequence of *Bacillus cereus* sequence type 2255 isolated from powdered infant formula

**DOI:** 10.1128/mra.00702-24

**Published:** 2024-09-17

**Authors:** Sonali Patil, Catherine W.Y. Wong, Richard Zhang, Annika Zhang, Xinyi Zhou, Wei Zhang

**Affiliations:** 1 Department of Food Science and Nutrition, Institute for Food Safety and Health, Illinois Institute of Technology, Chicago, Illinois, USA; 2 Hinsdale Central High School, Hinsdale, Illinois, USA; University of Maryland School of Medicine, Baltimore, Maryland, USA

**Keywords:** powdered infant formula, food safety, *Bacillus cereus*, genome

## Abstract

The draft genome sequence of a *Bacillus cereus* strain, DFPST-SP1, isolated from powdered infant formula in the United States is reported. The 5,216,828-bp draft genome comprises 46 contigs with 66.4× coverage and 36% GC content and was typed as sequence type 2255.

## ANNOUNCEMENT

Powdered infant formula (PIF) has been the subject of recalls in the United States in recent years due to possible contamination with foodborne pathogens (1, 2). Since PIF products are not sterile, a background population of microbiota is expected. Twelve different formulations of PIF available at retail in the Chicago, Illinois area were subjected to enrichment to identify the possible presence of foodborne pathogens as per the U.S. Food and Drug Administration (FDA) Bacteriological Analytical Manual (3–5). To assess for the presence of *Listeria monocytogenes* (5), triplicate 25 g samples of each formulation were homogenized with 225 mL of Buffered *Listeria* Enrichment broth (BLEB) and incubated statically at 30°C for 24 h; specific BLEB supplements were added after 4 h. After 24 h, enrichments were streaked onto Brilliance *Listeria* agar (BLA; Thermo Scientific, Waltham, MA, USA).

Green-blue colonies on BLA, generally typical of *Listeria* spp., but also some *Bacillus* spp., were observed from eight out of twelve PIF formulations. These colonies were subjected to whole-genome sequencing. Isolates were cultured in 5 mL of tryptic soy broth statically at 37°C for 18 h. Genomic DNA was extracted from the cultures using the DNeasy Blood and Tissue Kit (Qiagen, Germantown, MA, USA) according to the manufacturer’s instructions. The DNA quality and quantity were assessed using the Qubit Fluorometer 3.0 (Life Technologies, Waltham, MA, USA). The library for sequencing was prepared using the Illumina DNA Prep Kit (Illumina, San Diego, CA, USA) and sequenced on a MiSeq (Illumina) using 2 × 250 bp paired-end chemistry (500 cycles) with 5% PhiX (6).

While *Bacillus* spp. were identified in eight of the twelve PIF formulations, *B. cereus* was only identified in Formulation 1. The quality of the sequencing data (raw reads = 963,136, median insert = 271, and median length = 206.1) of the *B. cereus* isolate was checked using the GalaxyTrakr MicroRunQC workflow v 6 (7) and FastQC v 0.12.0 with default settings (8). A sequence coverage of ≥30× was required for read quality. The draft genome sequence was assembled using Skesa v 2.4.0 with default settings (9), and the genome completeness was assessed using CheckM v 1.1.6 with default settings (10). The genome sequence was annotated using the National Center for Biotechnology Information (NCBI) Prokaryotic Genome Annotation Pipeline v 6.6 (11, 12) with default settings and sequence typed as 2255 using the pubMLST 7-locus multilocus sequence typing (MLST) scheme for *B. cereus* (i.e., *glp*, *gmk*, *ilv*, *pta*, *pur*, *pyc*, and *tpi*) (6) (https://pubmlst.org/organisms/bacillus-cereus).

The draft genome sequence of the *B. cereus* strain contains 5,216,828 bp (212.5 Mb) composed of 46 contigs with an N_50_ of 289,806, a GC content of 36%, and a coverage depth of 66.4×. A total of 5,282 protein-coding genes, 86 putative RNA genes, and 74 tRNAs were predicted.

This announcement reports the draft genome sequence of a *B. cereus* isolate from powdered infant formula. No other foodborne pathogens were identified in the formulations tested.

## Data Availability

This Whole Genome Shotgun project has been deposited in NCBI, BioProject PRJNA1119532, BioSample SAMN39831649, Sequence Read Archive (SRA) accession no. SRR27873194 and GenBank accession no. JBAJIN000000000.
